# Principles of Tracer Kinetic Analysis in Oncology, Part II: Examples and Future Directions

**DOI:** 10.2967/jnumed.121.263519

**Published:** 2022-04

**Authors:** Austin R. Pantel, Varsha Viswanath, Mark Muzi, Robert K. Doot, David A. Mankoff

**Affiliations:** 1 Department of Radiology, University of Pennsylvania, Philadelphia, Pennsylvania; and; 2 Department of Radiology, University of Washington, Seattle, Washington

**Keywords:** kinetic analysis, dynamic imaging, PET/CT

## Abstract

**Learning Objectives:** On successful completion of this activity, participants should be able to (1) describe examples of the application of PET tracer kinetic analysis to oncology; (2) list applications research and possible clinical applications in oncology where kinetic analysis is helpful; and (3) discuss future applications of kinetic modeling to cancer research and possible clinical cancer imaging practice.

**Financial Disclosure:** This work was supported by KL2 TR001879, R01 CA211337, R01 CA113941, R33 CA225310, Komen SAC130060, R50 CA211270, and K01 DA040023. Dr. Pantel is a consultant or advisor for Progenics and Blue Earth Diagnostics and is a meeting participant or lecturer for Blue Earth Diagnostics. Dr. Mankoff is on the scientific advisory boards of GE Healthcare, Philips Healthcare, Reflexion, and ImaginAb and is the owner of Trevarx; his wife is the chief executive officer of Trevarx. The authors of this article have indicated no other relevant relationships that could be perceived as a real or apparent conflict of interest.

**CME Credit:** SNMMI is accredited by the Accreditation Council for Continuing Medical Education (ACCME) to sponsor continuing education for physicians. SNMMI designates each *JNM* continuing education article for a maximum of 2.0 AMA PRA Category 1 Credits. Physicians should claim only credit commensurate with the extent of their participation in the activity. For CE credit, SAM, and other credit types, participants can access this activity through the SNMMI website (http://www.snmmilearningcenter.org) through April 2025.

Kinetic analysis of dynamic PET imaging enables the estimation of biologic processes relevant to disease. Through mathematic analysis of the interactions of a radiotracer with tissue, information can be gleaned from PET imaging beyond static uptake measures. Part I of this 2-part continuing education paper reviewed the underlying principles and methodology of kinetic modeling. In this second part, the benefits of kinetic modeling for oncologic imaging are illustrated through representative case examples that demonstrate the principles and benefits of kinetic analysis in oncology. Examples of the model types discussed in part I are reviewed here: a 1-tissue-compartment model (^15^O-water), an irreversible 2-tissue-compartment model (^18^F-FDG), and a reversible 2-tissue-compartment model (3′-deoxy-3′-^18^F-fluorothymidine). Kinetic approaches are contrasted with static uptake measures typically used in the clinic. Overall, this 2-part review provides the reader with background in kinetic analysis to understand related research and improve the interpretation of clinical nuclear medicine studies with a focus on oncologic imaging.

In part I of this review (*1*), we illustrated the complex interactions in tissue that a PET radiotracer undergoes after injection, reflecting factors that mediate tracer delivery, retention, and release based on the cancer biology targeted by the tracer and its pharmacologic properties. By continuously imaging time course data of radiotracer uptake, retention, and washout and applying mathematic models to the time-varying 3-dimensional (4-dimensional) image dataset, PET and kinetic analysis can quantify tumor biology relevant to diagnosis and treatment guidance. In part I of this 2-part review, the underlying principles and methodology of kinetic modeling were discussed, including dynamic imaging protocols, model formulation based on tracer biology, kinetic parameter estimation, mathematic testing of a model, and graphical or simplified approaches. In this part II, we provide representative real-world examples of the principles outlined in part I.

Beyond an exercise in mathematics, the quantitation of a biologic process as measured by kinetic analysis can provide insight into the underlying biology. The true benefit of kinetic analysis lies in its application. Here, in part 2 of this review, representative examples of PET studies are discussed that exemplify cases whereby the interpretation of radiotracer uptake benefitted from kinetic analysis. A 1-tissue-compartment model (^15^O-water), an irreversible 2-tissue-compartment model (^18^F-FDG), and a reversible 2-tissue-compartment model (3′-deoxy-3′-^18^F-fluorothymidine [^18^F-FLT]) are reviewed in detail. Kinetic measures are contrasted with static approaches to illuminate the benefits of full kinetic analysis. The limitations of widely adopted static measures are also highlighted to guide the interpretation of routine static images as are typically obtained in the nuclear medicine clinic.

## ILLUSTRATIVE EXAMPLES

These representative examples are chosen to illustrate the application of the principles and methodology reviewed in part I of this continuing education review (*1*).

### 1-Tissue-Compartment Model

#### ^15^O-Water to Measure Blood Flow

Measures of tissue perfusion provide clinically important information in several contexts, most notably cardiology and neurology. Perfusion measures also provide insight into tumor biology and have been used for largely investigational biomarker applications in oncology. Although other tracers (e.g., ^82^RbCl) and other modalities (e.g., dynamic contrast-enhanced MRI and arterial spin labeling MRI) have been studied (*2*–*4*), ^15^O-water is a freely diffusible, inert radiotracer that, even though less clinically practical largely because of an approximately 2-min half-life, serves as a reference standard for perfusion imaging (*5*). The traced substance, water, diffuses freely from the capillaries into and out of a cell without trapping. Accordingly, a 1-tissue-compartment model characterizes this biology (Fig. 3 from part I (*1*)). The differential equation for a 1-tissue-compartment model is written below and can be solved for the variables of interest, blood flow and volume of distribution (*6*):

$$\frac{dC_{t}\left(t\right)}{dt}=F\cdot C_{a}\left(t\right)-\;(\frac{F}{V_{T}}+\lambda )\;(C_{t}\left(t)\right),$$

where *C_t_*(*t*) is the tissue concentration of tracer, *C_a_*(*t*) is the arterial activity, *F* is blood flow (=*K*_1_), and *V_T_* is volume of distribution (=*K*_1_/*k*_2_). This model can also directly account for physical decay of this short-lived isotope by including the ^15^O decay constant, **λ**, in the right side of the equation (boldface letter). We do note that if decay-corrected data are used, the decay constant can be omitted from the equation, and identical results will be achieved if appropriate weighting factors to account for frame length and delay are used. From PET images, both the arterial activity (e.g., an image-derived input function) and the tissue concentration of tracer can be measured so that blood flow and distribution volume may be solved, providing estimates of biologically relevant parameters. High blood-pool activity combined with rapid washout makes it challenging to use static uptake measures to estimate blood flow with this radiotracer. As such, the use of kinetic analysis is vital to image interpretation.

Blood flow imaging with ^15^O-water has been explored as a biomarker in the context of cancer blood flow, including in applications to breast cancer (*7*–*11*). In these studies, kinetic analysis of ^18^F-FDG PET studies accompanied ^15^O-water studies to study the ability of 2 radiotracers, each measuring different aspects of biology, to predict tumor behavior, including response to therapy. These studies are discussed further below.

#### ^18^F-Fluciclovine to Detect Biochemical Recurrence of Prostate Cancer

Similar to ^15^O-water, ^18^F-fluciclovine kinetics can be modeled with 1 tissue compartment and reversible transport. As a synthetic amino acid, ^18^F-fluciclovine enters the cell through bidirectional amino acid transporters but is neither metabolized nor incorporated into macromolecules (*12*) so uptake of this radiotracer tracks amino acid transport. Consequently, like ^15^O-water, the radiotracer washes out over time. As expected on the basis of this biology, a 1-tissue-compartment model fits the data well. Distribution volume was well estimated using both a 1-tissue-compartment model and a Logan plot, consistent with a reversible transport model for this radiotracer. A 2-tissue-compartment model that separated the extracellular and intracellular space into 2 tissue compartments was also tested but did not yield meaningful improvements in the quality of fit as judged by the Akaike information criterion (*13*). Reversible kinetics inform the clinical imaging protocol of ^18^F-fluciclovine in men with prostate cancer. Imaging begins at the pelvis 3–5 min after radiotracer injection and moves cranially so that peak lesional activity is captured in anatomic regions (the pelvis) most likely to harbor metastases. Since ^18^F-fluciclovine washes out, sensitivity for disease can decrease for imaging times late after injection. Early imaging to identify metastases with high target-to-background contrast differs from other clinical protocols, including ^18^F-FDG and ^68^Ga-DOTATATE, which are usually imaged at 60 min after injection to leverage trapping of the radiotracer (*14*–*16*). Likewise, the recently approved prostate-specific membrane antigen agents—^18^F-DCFPyL and ^68^Ga-PSMA-11—are both imaged at 1 h, reflecting (nearly) irreversible kinetics (*17*–*19*). In the clinic, detection of sites of disease in men with biochemical recurrence of prostate cancer with ^18^F-fluciclovine is largely qualitative, comparing uptake in suspected lesions with blood pool and marrow uptake. Given rapid radiotracer kinetics over the imaging interval, semiquantitative analysis—for example, SUV_max_—for prostate cancer is of limited utility, although such data may be given for reference (*20*). However, for other indications, such as imaging of gliomas, kinetic estimates or quantitative static uptake measures from later imaging may prove useful (*21*).

### Kinetic Analysis of ^18^F-FDG (2-Tissue-Compartment Irreversible Model), in Combination with ^15^O-Water, to Predict Outcome in Locally Advanced Breast Cancer

As detailed as the representative example in our companion paper discussing the principles and methodology of kinetic analysis, the biology of ^18^F-FDG requires modeling with a 2-tissue-compartment irreversible model in most tissues. From this model, kinetic parameters that estimate biologic processes of energy metabolism can be estimated, including ^18^F-FDG blood-to-tissue delivery (*K*_1_) and ^18^F-FDG flux (*K_i_*). Multiplying the *K_i_* (units of mL/min/cm^3^) by the measured plasma glucose concentration (μmol/mL) of a subject yields the metabolic rate of ^18^F-FDG (MR_FDG_), an approximation of glucose flux as estimated by ^18^F-FDG PET (plasma glucose concentration multiplied by *K_i_*), with resultant units in the form of μmol/min/cm^3^. Of note, a proportionality factor, the ^18^F-FDG lumped constant, is necessary to convert the MR_FDG_ to the metabolic rate of glucose (*22*), underscoring the known differences between glucose and ^18^F-FDG metabolism.

**FIGURE 1.**
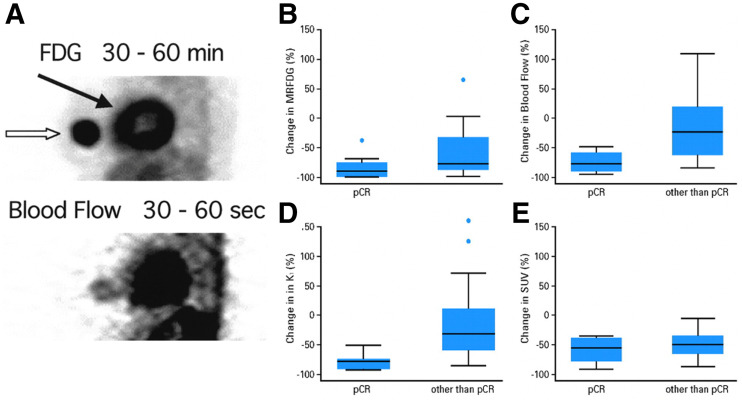
(A) Thick sagittal PET images of ^18^F-FDG (top) and ^15^O-water (bottom) demonstrate ^18^F-FDG uptake throughout breast cancer (open arrow), with relatively decreased blood flow centrally; solid arrow denotes heart. This regional metabolism–blood flow mismatch centrally suggests region of hypoxia. After chemotherapy, residual viable tumor was seen in center of tumor, suggesting chemotherapy resistance. (Reprinted from (*7*), noting that analysis in this publication used ROIs that did not account for tumoral heterogeneity.) (B–D) Changes in kinetic parameters (MR_FDG_ [B]; blood flow estimated by ^15^O-water [C]; ^18^F-FDG *K_i_* [D]) from baseline to mid therapy in study of patients with LABC demonstrate associations with tumor response. (E) Changes in SUV, however, were not significant. pCR = pathologic complete response. (Reprinted from (*11*).)

Kinetic analysis of ^18^F-FDG and ^15^O-water dynamic PET have been well explored as biomarkers for response in breast cancer, with kinetic analysis of both tracers demonstrating value (*7**,**10**,**23*). In these studies that leveraged dynamic imaging and kinetic analysis of sequential ^15^O-water and ^18^F-FDG dynamic PET, it was noted that, unlike normal breast tissue, the relationship between tumor glucose metabolism estimated by dynamic ^18^F-FDG PET and blood flow estimated by ^15^O-water was highly variable (*10**,**23*). Studies showed the utility of parameters quantifying the delivery of ^18^F-FDG (measured by the blood-to-tissue transport constant, *K*_1_) and its flux through the glucose metabolism rate-limiting step and hexokinase (measured by the flux constant, *K_i_*). In a study of women with newly diagnosed locally advanced breast cancer (LABC), patients with high MR_FDG_ relative to blood flow had a poor response to neoadjuvant chemotherapy. In this study, among many clinical, pathologic, and PET kinetic parameters, only the ratio of MR_FDG_ to blood flow, as assessed by ^15^O-water, demonstrated a significant difference for patients with versus without a macroscopic pathologic complete response to neoadjuvant chemotherapy (i.e., no macroscopic tumor seen on gross analysis of surgically resected tissue), a clinical endpoint with prognostic implications. A low ratio predicted response to chemotherapy. Alternatively, a high MR_FDG_-to-flow ratio, indicative of elevated glycolysis relative to flow such as would be seen with tumor hypoxia, portended a poor response to neoadjuvant therapy, corroborating independent observations supporting resistance of hypoxic tumors to chemotherapy (*7*). A representative example of this observation is shown in Figure 1A. In another study of untreated breast cancer patients, there was no correlation between estimates of blood flow from dynamic images of ^15^O-water versus an ^15^O-water SUV image from 4–6 min. This supports the concept that flow information cannot be captured in a late static SUV image, and kinetic analysis is required for this tracer with rapid washout (*8*). In addition to predicting treatment response, combined dynamic ^15^O-water and ^18^F-FDG PET revealed differences in the relationship between perfusion and glucose metabolism for different subtypes of breast cancer, providing insight into observed differences in patterns of treatment response in the clinic (*24*). These studies illustrate the clinical and biologic insights that can be gleaned from more detailed PET image acquisition and analysis.

Sequential dynamic imaging and kinetic analysis can also provide insights into therapeutic response. In a follow-up analysis of the aforementioned study with LABC patients (*7*), dynamic ^18^F-FDG and ^15^O-water PET studies were performed at both baseline and after 2 mo of chemotherapy. A decrease in blood flow between scans was seen in responders but not in nonresponders who had an average increase (−32% and +48%, respectively) (*9*). Patents whose tumor blood flow failed to decline with treatment had poorer disease-free and overall survival. Increased angiogenesis, possibly related to hypoxia, was hypothesized to explain these findings (*9*). Additional analysis of these patients demonstrated normalization of the metabolism–to–blood flow ratio after therapy, suggesting successful treatment of hypoxia (*10*). In analysis with an additional 18 patients, patients with persistent or elevated blood flow estimated by ^15^O-water and ^18^F-FDG *K*_1_ between baseline and the midpoint of neoadjuvant chemotherapy had higher rates of recurrence and mortality risks (*11*). Multivariate analysis controlling for known prognostic factors demonstrated that changes in blood flow and ^18^F-FDG *K*_1_ retained predictive ability for disease-free survival and overall survival; change in SUV was not predictive. Additionally, changes in kinetic parameters from baseline to mid therapy were significantly associated with tumor pathologic response, whereas change in SUV was not (Fig. 1B) (*11*). These results exemplify the added value of kinetic analysis.

Expanding the aforementioned ^18^F-FDG analysis, a second analysis of data from 75 LABC patients with dynamic imaging showed that the serial dynamic ^18^F-FDG alone, even without the water data, added key measures predictive of response. The study-observed changes in ^18^F-FDG *K*_1_ and *K_i_* predicted both disease-free survival and overall survival; changes in SUV predicted only overall survival (*25*). Only a change in *K*_1_ remained a significant predictor of overall survival when known prognostic factors were included in the model; a change in SUV was not significant (*25*). This study indicated that estimates of glucose delivery to tissue (^18^F-FDG *K*_1_) have value as a predictive marker of response and again underscored the benefits of kinetic measures over static measures.

#### Additional Applications of Blood Flow and ^18^F-FDG Kinetic Analysis

The ability of both ^18^F-FDG *K*_1_ and blood flow, as estimated by ^15^O-water, motivated studying ^18^F-FDG *K*_1_ as a proxy for flow, recognizing the difficulties and inherent challenges in regularly using ^15^O-water given its 2-min half-life. ^18^F-FDG *K*_1_, as discussed in part I, represents the delivery of radiotracer to tissue, inclusive of blood flow and transport across membranes. As such, this rate constant is not synonymous with blood flow. By the Fick principle, *K*_1_ can be approximated by blood flow multiplied by the first-pass extraction fraction of the tracer. For ^15^O-water, the extraction fraction is assumed to equal 1 so that ^15^O-water *K*_1_ equals blood flow (*26*). For tracers with lower first-pass extraction (e.g., ^18^F-FDG), the extraction fraction is less than 1, and *K*_1_ consequently does not equal blood flow. Nevertheless, there is a moderately strong correlation between blood flow as measured by ^15^O-water and ^18^F-FDG *K*_1_ (*10*). In a follow-on related approach, tumor blood flow has been estimated from the first pass of ^18^F-FDG using a 1-tissue-compartment model with data obtained during the first 2 min after injection (*27**,**28*). By analyzing such a short period after injection, the metabolic extraction fraction from ^18^F-FDG phosphorylation can be separated from the first-pass extraction fraction of ^18^F-FDG, which in turn can better estimate blood flow. In a study that included various tumor types, a correlation coefficient of 0.86 was found between measures of blood flow by the first pass of ^18^F-FDG and blood flow as estimated by ^15^O-water (*28*). Humbert et al. applied these methods and reported that blood flow changes were capable of stratifying patient groups with different overall survival percentages in women whose triple-negative breast cancer did not have a complete pathologic response (*29*). This approach could be implemented as a short flow-phase ^18^F-FDG PET scan early after injection, which could be practical in the clinic, akin to a 3-phase bone scan.

PET kinetic analysis can help inform the interpretation of dynamic contrast studies from other modalities. For example, blood flow estimated by ^15^O-water and ^18^F-FDG delivery (*K*_1_) have been correlated with dynamic contrast-enhanced MRI, a measure of tumor perfusion, noting that breast MRI plays a role in current diagnostic algorithms for breast cancer (*30*). Peak signal enhancement ratio, a measure of contrast washout in the tumor, correlated with blood flow and *K*_1_ (with each *r* > 0.7), suggesting a relationship between MRI contrast enhancement and blood flow. MR_FDG_ did not correlate with peak signal enhancement ratio, underscoring the different facets of biology queried with each modality (*31*). The association between these measures was also studied in LABC patients undergoing neoadjuvant chemotherapy. Changes in response to chemotherapy in ^18^F-FDG *K*_1_ correlated with changes in dynamic contrast-enhanced MRI signal enhancement ratio. Greater decreases in *K*_1_, *K_i_,* signal enhancement ratio, and peak enhancement were seen in patients with a pathologic complete response than in those without, suggesting utility in both modalities in predicting response (*32*). This finding also supports the use of a combination of MRI and ^18^F-FDG PET to predict and measure the response of LABC to neoadjuvant chemotherapy (*33*). In addition, blood flow by ^15^O-water has been shown to directly correlate with uptake of ^99m^Tc-sestamibi, a blood flow tracer used for both cardiac and breast cancer imaging, and inversely with ^99m^Tc-sestamibi washout (*34*). These findings suggest that, in tumors, both ^99m^Tc-sestamibi uptake and washout are influenced by blood flow, which should be considered in the interpretation of static breast ^99m^Tc-sestamibi images, such as those obtained for molecular breast imaging (*35*).

### Static Versus Kinetic Measures of ^18^F-FDG Uptake

**FIGURE 2.**
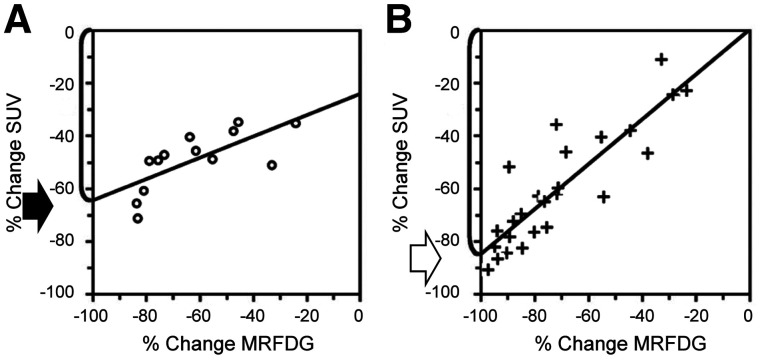
In study quantifying response to chemotherapy in breast cancer, percentage change in SUV is compared with percentage change in MR_FDG_. For patient in lowest tertile of baseline SUV uptake (A), only 65% of maximum detectable percentage change (solid arrow) in SUV (change in SUV when change in MR_FDG_ = −100%) is able to be theoretically achieved. This is compared with 86% of maximum detectable percentage change in SUV in patients with greater baseline uptake (open arrow) (B), underscoring impact of nonspecific uptake on static ^18^F-FDG uptake measures. (Adapted from (*36*).)

Static uptake measures, such as SUV, may serve as a proxy for kinetic measures and may have clinical relevance but do not directly estimate a specific biologic process. Rather, these static uptake measures represent the aggregate of many processes. In particular, static uptake measures cannot account for nonspecific radiotracer uptake, of particular importance when measuring response in tumors with low baseline uptake. For example, in a study of quantifying response to chemotherapy in LABC, a static SUV was compared with the MR_FDG_ (*36*). The percentage change in SUV versus that in MR_FDG_ from baseline to after therapy was analyzed for patients in the lowest tertile of baseline SUV uptake (SUV_mean_, 2.5; range, 1.6–3.0) compared with all others (SUV_mean_, 6.2; range, 3.1–12.3). The slope of the correlation for patients in the lowest tertile was significantly lower than for the other patients (0.4 vs. 0.85), indicating a falsely blunted assessment of response using SUV compared with MR_FDG_, particularly for subjects with low baseline uptake (Fig. 2). When the MR_FDG_ was extrapolated to −100%, indicating complete inhibition of ^18^F-FDG metabolism, the percentage change in SUV in the lowest tertile was 65%, compared with 86% in the other patients. The inability to distinguish nonmetabolized and trapped ^18^F-FDG in the static measure blunts the maximum detectable response and again underscores the limitations of using a static uptake measure as a proxy for a complex biologic process (*36*). These insights derived from kinetic modeling were corroborated in another clinical study in LABC patients with tumors larger than 3 cm monitored with ^18^F-FDG throughout therapy. If the pretherapy tumor-to-background ratio was less than 5, changes in ^18^F-FDG uptake from baseline were not predictive of tumor response; however, changes in patients with a tumor-to-background ratio of more than 5 were predictive (*37*). For these reasons, caution should be exercised when interpreting changes, or lack therefore, in ^18^F-FDG uptake in lesions with low baseline uptake in the clinic. These limitations in static measures may hamper the potential of these measures to serves as biomarkers, such as was exemplified in the study by Dunnwald et al. described above, in which kinetic measures were predictive of response in LABC but static measures were not for all response metrics (*25*).

The inherent limitations of static imaging, particularly the inability of static measures to account for nonspecific ^18^F-FDG uptake, are considered in imaging response criteria. For example, target lesions in PERCIST must have uptake greater than a threshold defined by background liver uptake, in large part to ensure the ability to detect a decrease in percentage radiotracer uptake with effective treatment (*38*). This understanding of the principles of kinetic analysis benefits the interpretation of even routine static images.

Kinetic analysis can avoid the pitfalls of measuring a dynamic process at a single time point with a static image and can even suggest that correction approaches could enhance static analyses. In a study of untreated breast cancers undergoing both dynamic and static ^18^F-FDG PET in a single session, ^18^F-FDG SUV_max_ changed linearly after 27 min, with both positive and negative slopes observed (range, from −0.02 to 0.15 SUV units/min). The rate of change of SUV also had a linear relationship with instantaneous SUV, and an empiric linear model to correct SUV for a variable uptake time was developed (*39*). Although this model demonstrated feasibility, such corrections are not used in routine clinical practice, and consensus recommendations suggest a consistent interval between injection and scanning (*40*).

The consequences of using static uptake measures on clinical trial design has been explored in virtual clinical trials. To explore the effect of variable uptake time, simulated ^18^F-FDG time–activity curves in women with LABC and static SUV measures were obtained at various time points in 4 distinct scenarios. These scenarios ranged from strict adherence to standardized uptake of 60–65 min to a combination of early and delayed scans with uptake times ranging from 45 to 115 min. Given that the ground truth of lesion uptake was known for any time point, the sensitivity and specificity of detecting a response to chemotherapy in breast cancer was studied. A sensitivity and specificity of 96% and 99%, respectively, was achieved in the scenario with highest compliance; this fell to 73% and 91%, respectively, for the least compliant group (*41*). Use of the correction algorithm above (*39*) improved both metrics. Simulated power analysis demonstrated that this variability increased sample sizes for simulated single-arm phase II trials (*41*). An additional study explored the effect of kinetic versus static measures on power or sample sizes for a virtual clinical trial. Sensitivity to detecting a response between a baseline and follow-up ^18^F-FDG PET scan was estimated for static uptake measures (SUV) and stratified by baseline uptake. As expected, larger sample sizes were required when static measures were used than when kinetic measures were used, and sample sizes were greatest for lesions with low baseline uptake. Sample size also decreased with better calibration of the PET scanners, underscoring the need for standardization in clinical trials, particularly in multisite clinical trials (*42*). In recognition of the variability of radiologic measures and the impact on biomarker development, the Radiological Society in North America established the Quantitative Imaging Biomarkers Alliance in 2007. A recent profile published by this alliance discusses many of these issues and provides claims on the precision of SUV measurements (*43*). The European Advanced Translational Research Infrastructure in Medicine serves as the European equivalent (*44*).

### Proliferation Imaging: ^18^F-FLT (2-Tissue-Compartment Reversible Model)

We discuss the analysis of images for ^18^F-FLT as a tracer with similar, but not identical, kinetics to ^18^F-FDG as a further illustration of the application of kinetic modeling to oncologic imaging. Radiolabeled thymidine and its analogs have been studied as markers of cellular proliferation, with increased rates of proliferation characteristic of malignancy (*45**,**46*). Through the exogenous salvage pathway, extracellular thymidine is incorporated into DNA, with the phosphorylation of thymidine by thymidine kinase I representing the initial and rate-limiting step. Because thymidine is incorporated into DNA, but not RNA, thymidine uptake reflects DNA synthesis and, thus, cellular proliferation (*45**,**47*).

Initial studies of ^11^C-thymidine demonstrated the ability to estimate cellular proliferation through kinetic analysis of this radiolabeled native analog. A 5-tissue-compartment model accounting for blood metabolites was able to estimate the flux constant accurately, though all model microparameters could not be estimated independently (*48**,**49*). The short half-life of ^11^C, combined with a complex analysis, precluded widespread use of this radiotracer, necessitating a different analog for clinical translation.

**FIGURE 3.**
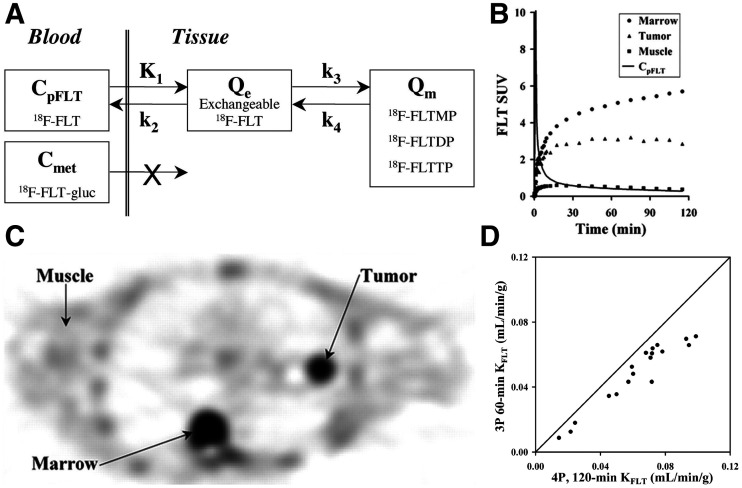
(A) Compartmental model of ^18^F-FLT with 2 reversible tissue compartments. (B) Representative time–activity curves for tumor, muscle, and marrow. (C) ^18^F-FLT-PET image demonstrating left lung cancer and normal marrow uptake. (D) Correlation of *K*_FLT_ from 3-parameter model using 60 min of data compared with 4-parameter model with 120 min of data shows underestimate of *K*_FLT_ with 3-parameter model using more data, as expected from preliminary mathematic studies. (Adapted from (*54*).) C_met_ = concentration of metabolites in arterial plasma; C_pFLT_ = concentration of ^18^F-FLT in arterial plasma; FLT-gluc = ^18^F-FLT-glucuronide; FLTDP = ^18^F-FLT-diphosphate; FLTMP = ^18^F-FLT-monophosphate; FLTTP = ^18^F-FLT-triphosphate; Q_e_ = exchangeable tissue compartment; Q_m_ = compartment of trapped ^18^F-FLT phosphorylated nucleotides.

The complexity of acquiring and analyzing ^11^C-thymidine PET images motivated the development of less heavily metabolized thymidine analogs as proliferation tracers (*45*). A fluorinated analog of thymidine, ^18^F-FLT, has advanced into clinical trials, benefitting from a longer half-life and fewer metabolites than for ^11^C-thymidine. Similar to ^11^C-thymidine, though, ^18^F-FLT traces the exogenous (salvage) thymidine pathway and can, as such, provide information on cellular proliferation similar to that from thymidine. However, unlike thymidine, ^18^F-FLT is not incorporated into DNA. Flux through the thymidine salvage pathway is ideated by retention of the ^18^F-FLT phosphorylated by thymidine kinase I, as the downstream product—^18^F-FLT-monophosphate or a related compound—is predominately trapped in the cell. Thus, like ^18^F-FDG, ^18^F-FLT is another largely trapped tracer that can be modeled with 2 tissue compartments (Fig. 3) (*50*).

However, several nuances for ^18^F-FLT necessitate considerations in the model that are not present for ^18^F-FDG. Metabolism of ^18^F-FLT by the liver produces ^18^F-FLT-glucuronide, which is restricted to the vascular space and contaminates the input function. This requires a metabolite-corrected input function in humans. Also, the washout rate from the trapped compartment (indicated by *k*_4_), related to dephosphorylation or transport of phosphorylated ^18^F-FLT (*51*), is more variable than it is for ^18^F-FDG (*50**,**52*). These factors were examined in a series of studies in both humans and animals (*50**,**52**,**53*). Simulation studies over a range of expected parameter values from clinical studies with 120 min of data demonstrated a 2-tissue-compartment reversible model with 4 rate constants, and a metabolite-corrected arterial input function accurately estimated ^18^F-FLT flux (*K*_FLT_ = (K_1_*k*_3_)/(*k*_2_ + *k*_3_)) and *K*_1_ (*r* = 0.99 and 0.94, respectively). In contrast, *k*_3_, representing the rate-limiting phosphorylation by thymidine kinase I, was not well estimated (*r* = 0.73), corroborating sensitivity and identifiability analysis (*50*). Using only the initial 60 min of data and eliminating *k*_4_, as suggested in earlier analyses (*53*), demonstrated −28% bias in *K*_FLT_. Such an underestimate may lead to incorrect conclusions in response studies, underscoring the importance of appropriate model selection and testing (*50*).

Validation studies in patients with lung cancer corroborated results from the mathematic simulation study. Compared with a 4-parameter model using 120 min of data, a 3-parameter model with 60 min of data underestimated *K*_FLT_, underscoring the need to account for dephosphorylation in this tissue type (Fig. 3). An SUV of 30–60 min demonstrated a poor correlation with *K*_FLT_ with 120 min of data (*r* = 0.62). Tissue correlation studies demonstrated a high correlation of *K*_FLT_ (ρ of 0.92 and 0.88 with 4 parameters and 120 or 90 min of data, respectively), with Ki-67, an in vitro assay of proliferation, validating the model as a marker of cellular proliferation. The correlation between Ki-67 and average SUV was lower, with a ρ of 0.65 (*54*). The inability to accurately estimate the microparameter *k*_3_ precludes direct correlation with Ki-67, also noting that Ki-67 is a protein marker of proliferation but not directly involved in the thymidine pathway, mitigating the utility for direct correlation (*55*). These detailed kinetic studies suggest that human translational studies with ^18^F-FLT should include detailed kinetic analysis before obtaining only simpler static measures (*47*).

After the above studies, a mouse study with subcutaneously implanted tumors supported the use of a 2-tissue-compartment model with reversible phosphorylation. These investigators concluded that scans at least 90 min in duration that include *k*_4_ are necessary if absolute quantification of *K*_FLT_ is needed. Correlation of dynamic PET measures with Ki-67 revealed a high correlation with *K*_FLT_, and *K*_FLT_ was estimated with better precision than *k*_3._ The correlation with SUV and Ki-67 was weaker (*52*). We do note that the macroparameter *K*_FLT_ [*K*_FLT_ = (*K*_1_*k*_3_)/(*k*_2_ + *k*_3_)] includes the microparameters *K*_1_ and *k*_3_ and is thus influenced by the transfer rate constant (*K*_1_, which is dependent on blood flow) and rate-limiting phosphorylation by thymidine kinase I (*k*_3_).

To facilitate translation into the clinic, there have been efforts to simplify the imaging protocol of ^18^F-FLT. A blood input function derived from 8 venous samples and a single sample at 60 min for metabolite analysis has been validated. An image-derived input function from the aorta also correlated with venous blood sampling (*56*). Additional work with a population-based input function combined with limited blood samples (as few as 3) have been used to estimate *K_i_,* which showed a good correlation with estimates using full arterial sampling, as well as a good correlation with Ki-67 (*57*). An image-derived input function has also been validated in patients with high-grade glioma patients, further suggesting clinically feasible protocols (*58*). As detailed, kinetic measures have been shown to better correlate with Ki-67. Nonetheless, obtaining kinetic parameter estimates requires dynamic scanning and, in this case, metabolite correction. Moreover, in a reproducibility study in non–small cell lung cancer, kinetic measures (Patlak analysis and 2-tissue-compartment analysis with *k*_4_ = 0) with 60 min of dynamic data were less reproducible than static measures (*59*). Ultimately, the need for practical reproducible clinical protocols must be balanced with the ability of static uptake measures to capture relevant biology to improve clinical care.

### Future Direction: Whole-Body Scanners

**FIGURE 4.**
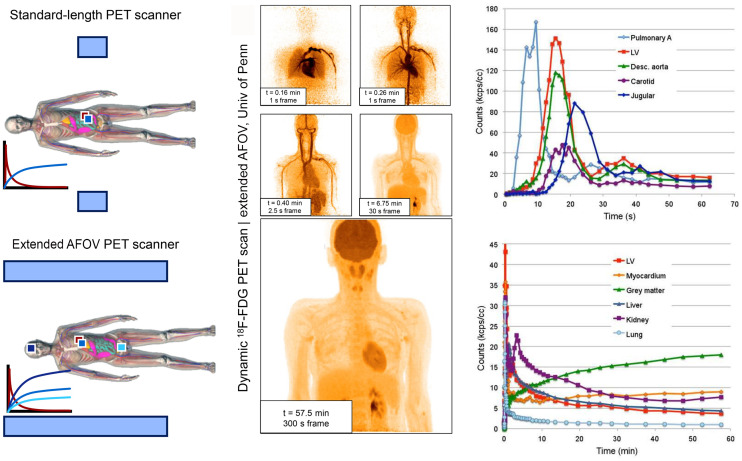
(Left) Schematic illustrating benefit of extended AFOV total-body (TB) PET scanners (blue rectangles represent AFOV of each scanner). Extended AFOV TB PET scanners enable simultaneous kinetic analysis of all major body organs. Images (middle) and time–activity curves (right) from dynamic ^18^F-FDG dataset of healthy human subject imaged on the extended AFOV scanner at the University of Pennsylvania demonstrate ability to capture relatively noise-free time–activity curves. (Adapted from (*62*).) Univ of Penn = University of Pennsylvania.

Although dramatic improvements in PET technology have revolutionized PET imaging, kinetic analysis applications, particularly in oncology, remain hampered by the limited axial field of view (AFOV) of modern PET scanners (<30 cm). To realize the full potential of PET imaging, long-AFOV PET scanners have been developed. The increased axial coverage of these instruments enables data collection from the entire burden of disease across the patient while simultaneously imaging a large blood vessel from which the image-derived input function can be measured without significant partial-volume effects. The 2-m total-body (TB) PET scanner at the University of California Davis (*60**,**61*) images the entire body in a single field of view; the TB PET scanner at the University of Pennsylvania can capture all major organs of the body in a single bed position (Fig. 4) and has recently been expanded from an AFOV of 1.12 m to one of 1.36 m (*62*–*64*). Additionally, the marked sensitivity gains of these instruments also enable relatively noise-free time–activity curves, as shown in Figure 4, in which early frames are 1 s in duration, particularly for the image-derived input function, for which short time bins may be used early in imaging (*63*). With advanced reconstruction methods on a TB PET scanner, a 100-ms temporal resolution was achieved (*65*). These sensitivity gains can be leveraged to image radiotracers at lower doses while maintaining accuracy of kinetic parameter estimation (*66*), of particular importance for new radiotracers with production challenges or an elevated organ dose. Imaging at lower doses may also be leveraged for dual-tracer imaging of 2 fluorinated radiotracers in a single imaging session (*67*), where the first radiotracer is injected at a markedly lower dose, minimizing residual activity during the second tracer acquisition, followed by a higher dose of a second tracer (*68*). Lastly, the inclusion of all major organs in the long AFOV enables whole-body kinetics to study the dynamic interactions between organs (*69*). With increased count statistics, these approaches may include a fit of the blood input curve and not just its use as a driving input function.

## CONCLUSION

In this second part of this 2-part continuing education review, the benefits of kinetic analysis of PET data were explored through representative case examples. Representative 1-tissue-compartment and reversible or irreversible 2-tissue-compartment models were reviewed to demonstrate the application of the principles and methodology discussed in part I. As demonstrated here, a kinetic model must be designed to estimate biologically relevant processes in an accurate and reproducible manner. Kinetic measures can avoid many of the pitfalls of using static measures to characterize a dynamic process as illustrated by the selected examples discussed in part II of this review. Although dynamic imaging for kinetic analysis is often impractical for the clinic, and many of the examples focus on research applications and questions, the concepts of tracer kinetics and kinetic analysis apply to the interpretation of static images for clinical oncologic PET imaging, including ^18^F-FDG, and should be considered in clinical image interpretation.
